# Supplementary material for: Mechanism for controlled assembly of transcriptional condensates by Aire
Source: Nat Immunol. 2024 Aug 21;25(9):1580–92. doi: 10.1038/s41590-024-01922-w (PMC11362013; doi:10.1038/s41590-024-01922-w)
Supplement: Supplementary file 1 — Supplementary Note. [file 41590_2024_1922_MOESM1_ESM.pdf]

nature immunology

Supplementary information https://doi.org/10.1038/s41590-024-01922-w

Mechanism for controlled assembly of transcriptional condensates by Aire

In the format provided by the authors and unedited

Supplementary Note

Expression vectors

Throughout the Supplementary Note, Aire indicates human Aire, unless mentioned otherwise. Generation of pInducer20-Aire-FLAG WT and C311Y, pCDNA3.1-Aire-FLAG along with truncation variant aa 106-545 (Aire ΔCARD), pEGFP-N1-mouse Aire-FLAG (EGFP gene removed) WT along with truncation variant aa 108-552 (mouse Aire ΔCARD) and pEGFP-N1-mouse Aire aa 1-173 fused to monomeric enhanced GFP (mGFP) with a 3XFLAG-TEV protease cleavage site linker between the mouse Aire NLS and mGFP (CARD-mGFP) were previously described[1]. Lentiviral vectors pMD2.G (encoding VSV-G), psPAX2 (encoding HIV Gag/Gag-Pol, Rev, and Tat), and pInducer20-GFP (encoding both tetracycline repressor and GFP downstream of a tetracycline response element) were generous gifts from Dr. Hidde Ploegh (Harvard Medical School; Boston, MA). All point mutations in this study were generated by using Phusion® High Fidelity DNA polymerase (New England Biolabs). The same mutagenesis strategy was used to introduce a stop codon and FLAG tag after aa 480 to generate pEGFP-N1 mouse Aire-FLAG Δaa 480-552 (ΔCTT). To generate pInducer20-Aire-FLAG K83E and G228W, point mutations were first incorporated within pCDNA3.1-Aire-FLAG and then DNA encoding Aire-FLAG point mutants were subcloned into pInducer20-GFP (GFP gene removed). Generation of all internal amino acid deletions of Aire plasmids were generated by inverse PCR of template plasmid using PrimeSTAR Max DNA polymerase (Takara Bio), followed by DpnI (New England Biolabs) template digestion, 5' phosphorylation and subsequent ligation of PCR products with T4 protein kinase and T4 DNA ligase, respectively (New England Biolabs). Aire Δaa 295-343 (ΔPHD1), Δaa 482-545 (ΔCTT), Δaa 499-509 (ΔCTT.R1), Δaa 510-521 (ΔCTT.R2), Δaa 522-535 (ΔCTT.R3) were first subcloned into pCDNA 3.1; subsequently, DNA encoding these Aire-FLAG variant constructs replaced DNA encoding GFP in pInducer20-GFP. DNA encoding APEX2 (cDNA derived from Addgene plasmid #49386[2], a gift from Dr. Alice Ting) and Myc NLS-mouse Aire aa 486-552 was subcloned into pCDNA3.1. pGL4.31 (Firefly luciferase reporter plasmid under 5XUAS box promoter) was a kind gift from Dr. George Church (Harvard Medical School; Boston, MA). phRLCMV (Renilla luciferase expression plasmid under CMV promoter) was a kind gift from Dr. Diane Mathis (Harvard Medical School; Boston, MA). DNA encoding GAL4 DNA binding domain (DBD) was amplified from pMBD-Gate2, a kind gift from Kamil Onder (Addgene plasmid # 25947)[3] and subcloned into pFLAG-CMV4 (pFLAG-CMV4-GAL4). mouse Aire aa 491-552 (CTT) WT, point mutants along with deletion variants Δaa 503-513 (ΔCTT.R1) Δaa 514-525 (ΔCTT.R2) and Δaa 526-539 (ΔCTT.R3) were subcloned into pFLAG-CMV4-GAL4. lentiCas9-Blast was a gift from Feng Zhang (Addgene plasmid # 52962)[4]. pLN426 (FuGW-G5p-mKate2) was a gift from Timothy Lu (Addgene plasmid # 105183)[5]. EGFP-P2A-Gal4DBD-mouse AireCTT was subcloned into pInducer20. FLAG tag, mouse Aire aa 486-552 (CTT construct used only for mass spectrometry) along with mouse Aire aa 503-552-FLAG (CTT-FLAG) WT, point mutants and deletion variants were subcloned into a modified pGEX-6P-1 vector containing a 6XHis-tag N-terminal to the GST tag. mouse Aire-FLAG point mutants and domain deletion variants Δaa 198-264 (ΔSAND), Δaa 301-342 (ΔPHD1), Δaa 433-475 (ΔPHD2), ΔCARDΔCTT, ΔSANDΔCTT, ΔPHD1ΔCTT, ΔPHD2ΔCTT, ΔSANDΔPHD2 (for clarity referred to as CARD-PHD1-CTT), ΔSANDΔPHD2ΔCTT (for clarity referred to as CARD-PHD1), and aa 1-290 (CARD-SAND) were subcloned into pEGFP-N1 (EGFP gene removed). pEGFP-N1 mouse Aire CARD-PHD2 was generated by replacing the DNA encoding for mGFP with DNA encoding mouse Aire PHD2 (aa 433-475) in pEGFP-N1 CARD-mGFP. pSG5-HA-p300 was a gift from Elizabeth Wilson (Addgene plasmid # 89094)[6]. Generation of HA-p300 truncation variants (amino acid boundaries listed in Extended Data Fig. 4d-f) involved the same inverse PCR strategy as described above. For mGFP-p300 variant fusions, p300 variants were subcloned into a modified pEGFP-N1 vector where A206K point mutation was introduced in enhanced GFP to encode monomeric GFP. pET22b-HisGb1-STAT1(aa 710-750) + CBP TAZ2 (aa 1764-1855) was a gift from Peter Wright (Addgene plasmid # 99342)[7]. mouse Aire CTT (aa 480-552) and mouse Aire PHD1 (aa 295-349) were subcloned into pET47b. DNA encoding CHD4 PHD2 (aa 439-493) was synthesized by Integrated DNA Technologies (IDT); this cDNA was used

as a template for subcloning into pET47b. cDNA for Sp110 CARD (aa 6-110) was amplified from MegaMan Human Transcriptome Library (Stratagene). The In-Fusion HD assembly method (Takara Bio) was used to generate CHD4-mouse Aire chimeras along with Sp110 CARD fusions with mGFP and mouse Aire PHDs within pEGFP-N1.

4D6 stable cell line generation
Lentivirus was first produced in 293T cells. 293T cells were seeded in a 12-well plate format and each well was transfected with 0.75 μg pInducer20, 0.33 μg psPAX2, and 0.18 μg pMD2.G using Lipofectamine 2000. 16 hours later, the medium was replaced. 48 hours after transfection, the medium (inoculum) was harvested and passed through a 0.45μm filter. For transduction, 4D6 cells were seeded in a 12-well plate. When cells were 70% confluent, the medium was replaced with a mixture of 300 μl filtered inoculum + 3 μl polybrene (10 mg/ml stock concentration, Sigma-Aldrich). Cells were incubated in the inoculum mixture with manual gentle agitation every 15 minutes for 1 hr. 700 μl of RPMI supplemented with 10% FBS, 1% L-glutamine was then directly added to each well with cells. 24 hours later, the transduced cells were trypsinized and transferred into a T25 flask to recover from transduction. After 24 hrs of recovery, 1 mg/ml G418 sulfate (Corning) was used for selection of transduced 4D6 cells. During G418 selection, medium was changed every 2-3 days. After the mock-transduced cells were ~95% dead, cells undergoing G418 selection were diluted into 96-well plates for individual clone selection.

Chromatin immunoprecipitation (ChIP)-seq and analysis (continued)
See Methods for experimental details of crosslinking of cells and harvest. When flash frozen cells were ready to use, cells pellets were thawed on ice for 15 minutes and then resuspended in ice-cold One-Step Lysis Buffer (50 mM Tris H 7.5, 1% SDS, 0.25% Sodium Deoxycholate and 1 X mammalian protease inhibitor cocktail). Cells were incubated on ice for 10 min and then 700 μl ChIP Dilution Buffer (50 mM Tris pH 7.5, 0.01% SDS, 150 mM NaCl, 0.25% Triton-X, 1 X mammalian protease inhibitor cocktail) was added. Chromatin was sheared using with a Covaris M220 ultrasonicator (settings: 5% duty factor, 75W max power, 200 cycles per burst, 20 min) at 6°C. Lysates were spun down in a refrigerated centrifuge for 10 minutes at 18,000 g. Cleared lysate was 3-fold diluted with ChIP Dilution Buffer, spike-in chromatin (600ng per pull-down, Active Motif) was added when cells were treated with p300 inhibitors or DMSO, and 2-3% of the input was saved for later use. Lysates were nutated with antibodies [for each pull-down when indicated, 2.5 μg of anti-FLAG (M2, Sigma); 5 μg anti-H3K4me1, 5 μg anti-H3K27me3, 4 μg of anti-p300 (D2X6N, Cell Signaling Technologies); 4 μg of anti-H3K27Ac (Cell Signaling Technologies); 4 μg of anti-H3K4me0 (Active Motif); an additional 2 μg spike-in antibody (Active Motif) was included when cells had been treated with p300 inhibitors or DMSO] for 16 h at 4°C.

Protein-DNA complexes were immunoprecipitated using Protein G magnetic beads (Active Motif) with 2 h nutation at 4°C. Protein G beads were washed with the following ice-cold buffers: RIPA buffer (0.1% SDS, 0.1% Sodium Deoxycholate, 1% Triton X-100, 1 mM EDTA, 10 mM Tris pH 8, 150 mM NaCl); RIPA supplemented with 350 mM NaCl; LiCl Buffer (10 mM Tris pH 8, 250 mM LiCl, 0.5% Triton X-100, 0.5% Sodium Deoxycholate) and Tris Buffer (10 mM Tris pH 8.5). Protein-DNA complexes were eluted with Elution Buffer (10 mM Tris pH 8, 1 mM EDTA, 0.1% SDS, 150 mM NaCl, 5 mM DTT) with gentle agitation at 65°C for 1 h. Reserved ChIP inputs were diluted two-fold with Elution Buffer. Dilute inputs and eluted protein-DNA were treated with RNAse (Machery-Nagel) at 37°C for 30 min, then Proteinase K (New England Biolabs) at 65°C for 16 h to ensure reverse crosslinking of DNA. Reverse cross-linked DNA was purified using SPRI Select beads (Beckman). ChIP-seq DNA libraries were prepared using NEBNext Ultra II DNA library Prep Kit for Illumina (New England Biolabs) according to the manufacturer's protocol. Deep sequencing was performed using a NovaSeq sequencer (Illumina) with pair-end 150 bp reads.

QC was performed on demultiplexed sequencing files using FASTQC (v0.11.3). Sequencing reads were trimmed using Trimmomatic (v0.36) and aligned to reference genome (GRCh38 primary

assembly) using bwa (v0.7.17). The resulting SAM files were converted to BAM files, sorted and indexed using Samtools (v1.6). Samples with drosophila spike-in chromatins were aligned to a customized reference genome consisting both hg38 and dm6, and the sorted and index BAM files were subsequently split into separate BAM files containing hg38- and dm6-mapped reads, respectively, using plit_bam.py module in the SPIKER tool[8]. hg38- and dm6-mapped reads were deduplicated using the alignmentSieve function in deepTools (v3.5.1), and unique dm6-mapped reads were counted to calculate the scaling factors. Peak calling was performed using MACS2 (v2.2.7.1)[9] with the following parameters: macs2 callpeak -f BAMPE -B -g 3.2e+9 --keep-dup 1 --SPMR --nomodel --extsize 250 -q 0.05 --cutoff-analysis. When calling peaks in WT and mutant Aire ChIP-seq samples, corresponding input controls were included to remove the effect of background noise. WT and mutant Aire ChIP-seq BAM files and peaks were imported into DiffBind (v3.8.4)[10], and a census of all Aire peaks (n=13023, summits +/– 200 bp, Supplementary Table 5) were obtained after removing blacklisted and greylisted regions using dba.blacklist and dba.peakset functions. Trimmed Mean of M-values (TMM)-normalized counts-per-million (CPM) reads[11] within each Aire peak (summits +/– 200 bp) were obtained using the dba.count function in DiffBind with options: bRemoveDuplicates = T, score=DBA_SCORE_TMM_MINUS_FULL_CPM. TMM-normalized CPM reads of H3K27ac ChIP and p300 ChIP around Aire peaks (Aire peak summits +/– 2000 bp for H3K27ac and summits +/– 200 bp for p300) were obtained with option score=DBA_SCORE_TMM_READS_FULL_CPM. Statistical comparison and significance were calculated using the Wilcoxon rank sum test in R. Spearman's correlation coefficient was calculated in Prism (Graphpad). Peaks called from H3K27ac ChIP-seq (–Aire expression) were removed of blacklisted regions and subjected to Ranking Ordering of Super Enhancer (ROSE) analysis[12] to obtain putative super-enhancer regions, using 12.5 Kb stitching distance and 2 Kb TSS exclusion parameters. BAM files were converted to bigwig files using deepTools (v3.5.1). Bigwig files for WT and mutant Aire ChIP-seq samples shown in Figs.1d, 4a, 4c, 6d and Extended Data Figs. 1a, 1f were generated by subtracting background noises in corresponding input controls with the following settings: bamCompare -b1 "pulldown bam file" -b2 "input bam file" -o "bw file" --operation subtract --scaleFactorsMethod None --normalizeUsing CPM --ignoreDuplicates -bs 50 -e 200 --smoothLength 150. Bigwig files for all other sequencing samples were generated without input controls using the bamCoverage function with the same setting options. After verification of consistency between replicates, bigwig files were averaged using WiggleTools (v1.2.2) and bedGraphToBigWig (v366) and imported into IGV (v2.15.1) for visualization at specific loci or into deepTools (v3.5.1) for generation of heatmaps and average profiles using the computeMatrix, plotHeatmap and plotProfile functions. The heatmaps shown in Figs. 4a and 6d were centered on the census of all Aire ChIP-seq peak-defined regions (n = 13023, +/– 2 Kb) and ranked based on the ratio of signals in WT versus ΔCTT.R3 samples. The heatmaps shown in Figs. 4f-g and Extended Data Fig. 1a were centered and scaled at the H3K27ac-delimited super-enhancer regions (n = 529, +/– 200 Kb) and ranked based on the ROSE ranking order.

Immunofluorescence with RNA-FISH analysis

Nascent RNA-FISH spots and Aire foci were identified in individual z-stacks using DiAna ImageJ plug-in[13]. For images generated from the same experiment, the "threshold" parameter for a given fluorescent channel was set the same across different groups, and the "min pixel" parameter was set to 20 for RNA-FISH spots and 5 for Aire foci. The distances between Aire foci and RNA-FISH spots or randomized nuclear spots were then measured using DiAna ImageJ plug-in[13]. Statistical comparison and significance were calculated in Prism. While RNA-FISH and Aire condensates often did not show concentric colocalization (Extended Data Fig. 2d), recent microscopy studies indicate that active genetic elements can partially overlap (within ~1 μm), rather than completely overlap with target genes[14]. The close association between Aire condensates and the target gene RNA-FISH foci therefore is consistent with the notion that Aire condensates directly activate transcription.

The average Aire signal at RNA-FISH spots was computed and plotted in MATLAB as previously described[15]. Briefly, the MATLAB scripts were obtained from github (https://github.com/krishna-shrinivas/FISH_IF_colocalization), and a list of RNA FISH signal centroids

(x, y, z) manually curated using DiAna from the previous step were provided to the MATLAB pipeline. The Aire IF signal centered at FISH spots were then combined to calculate an average intensity projection within a 2.8 x 2.8 μm^2 square. The same process was carried out for FISH signal centered on its own (x, y, z) coordinates. Spearman correlation coefficient (r_s) was computed and reported between the FISH and IF signals centered at FISH spots. As a control, the same process was carried out for Aire IF signal centered at random nuclear positions that were selected using this MATLAB pipeline. The average intensity projections were then used to generate 2D contour plots of the signal intensity. The averaged IF signal centered at FISH spots or randomly selected nuclear positions were plotted using the same color and intensity scale.

Chromatin fractionation assay
293T cells transfected with plasmids expressing mouse Aire were harvested and washed in PBS, then lysed with Hypotonic Buffer as described above. Nuclear fractions were then resuspended in Nuclear Extraction Buffer (50 mM Bis-Tris pH 7.5, 750 mM 6-aminocaproic acid, 3 mM $CaCl_2$, 10% glycerol, 1X mammalian protease inhibitor cocktail; 200 µl/sample) and split into fractions with or without MNase (Promega, 50U/100 µl of nuclear lysate) and incubated for 1 hour at 4°C. MNase activity was quenched with 5 mM EDTA. Nuclear lysates were centrifuged at 18,000 g for 10 minutes at 4°C. The resulting supernatant was the "soluble" nuclear fraction and saved for analysis. The "insoluble" nuclear pellet was washed one time with ice-cold PBS, then resuspended in Laemmli sample buffer and boiled for 5 minutes. The soluble and insoluble nuclear fractions were run on SDS-PAGE gel and subsequently analyzed by western blotting.

Immunofluorescence microscopy analyses
All quantitative immunofluorescence imaging analyses were performed with Fiji Is Just ImageJ (FIJI, ImageJ2 v2.14.0/1.54f). For each image z-stack, masks were drawn around nuclei based on DAPI fluorescence. Only cells that expressed Aire (anti-FLAG immunostaining) as visualized by nuclear fluorescence staining were analyzed further. For the calculation of mean fluorescence intensity per segmented nucleus, background subtraction was performed on each image to compare intensities between samples. To examine the number of Aire foci per nucleus, Aire foci were segmented using the Yen Dark thresholding method[16] featured in FIJI. For Aire foci volume analyses of 4D6 cells, the DiAna ImageJ plug-in[13] was used to 3D segment Aire foci and calculate Aire foci volumes using the same intensity thresholds when comparing samples.

To identify nuclei that contained Aire foci or "diffuse" Aire, image z-stacks were first manually inspected for the most in-focus 2D z-slice to further analyze. Within 2D images, a nucleus would be defined to have Aire foci with the presence of 2 or more circular spots (> 3 $pixels^2$) with maximum intensities >2 fold higher than background intensities within the nucleus. Diffuse nuclear staining was defined as uniform Aire fluorescence intensities throughout a given nucleus.

For image analyses of stable 4D6 cells expressing Aire-FLAG WT or ΔPHD1 co-stained with co-activators, image z-stacks were first manually inspected for the most in-focus 2D z-slice to further analyze. To identify Aire foci in a 2D image, an iterative implementation of Minimum Error intensity thresholding was used to create region of interests (ROIs) outlining Aire foci for a given nucleus. For every identified Aire focus, the average intensity signal from the corresponding ROI in the co-activator fluorescence channel was determined. The average intensities were normalized to the average co-activator intensity within the entire nucleus being examined. Statistical significance comparisons were calculated by using a Mann-Whitney test for two population proportions where each population consists of all individually normalized mean intensities of co-activators within the corresponding locations of Aire foci.

E. coli. expression and purification of recombinant proteins
His_6-GST, His_6-GST-FLAG, His_6-GST-CTT, His_6-GST-CTT-FLAG WT and variants were expressed in BL21(DE3) Rosetta (Millipore Sigma) cells at 37°C in Luria Broth (LB), grown to an OD_{600} ~0.8-1, then

induced with 0.4 mM IPTG for 3 h at 37°C. His_6-mouse Aire PHD1 and His_6-CHD4 PHD2 were expressed in BL21(DE3) Rosetta cells at 37°C in LB, grown to an OD_{600} ~0.5-0.6, then cooled down to 25°C for 40-60 minutes while still shaking, supplemented with 50 μm $ZnCl_2$ and induced with 0.4 mM IPTG for 5-6 hrs. His_6-STAT1(710-750) + CBP TAZ2 (1764-1855) were expressed in BL21(DE3) Rosetta cells at 37°C in LB, grown to an OD_{600} ~0.8-1, then cooled down to 15°C for 15-20 min minutes on ice, supplemented with 150 μm $ZnCl_2$ and induced with 0.4 mM IPTG for 16 hrs. His_6-mouse Aire CTT-FLAG was co-expressed with pCDF-GroEL/ES+trigger factor (a generous gift from Timothy A. Springer lab, Boston Children's Hospital; Boston, MA) in BL21(DE3) cells at 37°C in M9 minimal medium supplemented with $^{15}NH_4Cl$ and $^{13}C_6$-glucose; cells were grown to OD_{600} ~0.8 and cooled down to 25°C, then induced with 0.5 mM IPTG for 5 hrs.

Cells expressing His_6-GST and His_6-GST-CTT for mass spectrometry were harvested and resuspended in MS Lysis Buffer (50 mM Tris pH 8, 300 mM NaCl and 10% glycerol, 1 mM PMSF). Cells expressing His_6-GST-FLAG, His_6-GST-CTT-FLAG variants, His_6-mouse Aire PHD1, His_6-CHD4 PHD2 and $^{15}N^{13}C$-labeled His_6-mouse Aire CTT were harvested and resuspended in FP lysis buffer (50 mM HEPES pH 7.5, 200 mM NaCl, 5% glycerol, 1 mM PMSF). Cells expressing STAT1(710-750) + CBP TAZ2 (1764-1855) were resuspended in TAZ2 lysis buffer (50 mM Tris pH 8, 400 mM NaCl, 10 mM $MgCl_2$, 1 mM PMSF) supplemented with 10mM imidazole. All resuspended cell pellets were frozen and stored at -20°C until ready for purification. All protein purification procedures were performed at 4°C. All thawed cells were lysed with an Emulsiflex C3 (Avestin) and centrifuged at 32,000 g for 30 minutes. For each protein, cleared lysate was loaded onto a Ni^{2+}-NTA agarose (Qiagen) gravity-flow column.

For purification of His_6-GST and His_6-GST-CTT, the Ni^{2+}-NTA agarose columns were washed with 100 column volumes of MS Wash Buffer (50 mM Tris pH 8, 300 mM NaCl, 25 mM imidazole) and purified protein was eluted with 50 mM Tris pH 8, 300 mM NaCl and 50 mM - 250 mM imidazole. Imidazole elutions containing >95% pure protein were pooled and buffer exchanged back into MS Lysis Buffer using Amicon Ultra - 15 concentrators (Millipore) until ready for further use.

For purification of His_6-GST-FLAG, His_6-GST-CTT-FLAG variants, His_6-mouse Aire PHD1 and His_6-CHD4 PHD2, and $^{15}N^{13}C$-labeled His_6-mouse Aire CTT, the Ni^{2+}-NTA agarose columns were washed with 7 column volumes of FP Wash Buffer (50 mM HEPES pH 7.5, 300 mM NaCl, 25mM Imidazole). Purified protein was eluted with 50 mM HEPES pH 7.5, 300 mM NaCl and 50 mM - 150 mM imidazole. Imidazole elutions containing >90% pure protein were pooled and supplemented with 5 mM BME. His_6-GST-FLAG, His_6-GST-CTT-FLAG variants, and His_6-CHD4 PHD2 were dialyzed in FP Dialysis Buffer (25 mM HEPES pH 7.5, 200 mM NaCl, 5 mM BME) for 16 hrs. Dialyzed His_6-GST-FLAG and His_6-GST-CTT-FLAG variants were further purified with a Superdex 200 Increase 10/300 GL column (Cytiva) with SEC Buffer (25 mM HEPES pH 7.5, 100 mM NaCl, 5 mM BME). Dialyzed His_6-CHD4 PHD2 was buffered exchanged into HEPES Loading Buffer (25 mM HEPES pH 8, 25 mM NaCl, 5 mM BME), then loaded onto a HiTrap Q FF 1 ml column (Cytiva) and purified with a gradient (Buffer A: 25 mM HEPES pH 8, 5 mM BME; Buffer B: 25 mM HEPES pH 8, 1 M NaCl, 5 mM BME). Dialyzed $^{15}N^{13}C$-labeled His_6-Aire CTT was buffered exchanged into Tris Loading Buffer (25 mM Tris pH 8, 25 mM NaCl, 5 mM BME), then loaded onto a Resource Q 1 ml column (Cytiva) and purified with a gradient (Buffer A: 25 mM Tris pH8, 5 mM BME; Buffer B: 25 mM Tris pH 8, 1 M NaCl, 5 mM BME). Ion-exchange pooled fractions of His_6-CHD4-PHD2 and $^{15}N^{13}C$-labeled His_6-Aire CTT, and His_6-Aire PHD1 were further purified with a Superdex 75 Increase 10/300 GL column (Cytiva) with SEC Buffer. Purified His_6-GST-FLAG, His_6-GST-CTT-FLAG variants, His_6-Aire PHD1, and His_6-CHD4 PHD2 were concentrated using Vivaspin 2 concentrators (Sartorius), flash frozen in liquid nitrogen and stored at -80°C until ready for further use. $^{15}N^{13}C$-labeled His_6-Aire CTT was concentrated and dialyzed into NMR Sample Buffer (10 mM $NaPO_4$ pH 6.3, 30 mM NaCl, 1 mM DTT).

For His_6-STAT1(710-750) + CBP TAZ2 (1764-1855), cleared lysate and Ni^{2+}-NTA agarose beads were nutated for 2 hrs. Beads were then washed with 10 column volumes of the following: TAZ2 lysis buffer supplemented with 10 mM Imidazole; TAZ2 lysis buffer supplemented with 40 mM Imidazole; 50 mM Tris pH 8, 1 M NaCl; and 50 mM Tris pH 7.5, 500 mM NaSO4. His_6-STAT:CBP complex was eluted

with 50 mM Tris pH 7.5, 600 mM Imidazole. was buffered exchanged into MES Loading Buffer (25 mM MES pH 7, 50 mM NaCl, 5 mM BME), then loaded onto a Resource S 1 ml column (Cytiva) and purified with a gradient (Buffer A: 25 mM MES pH 7, 5 mM BME; Buffer B: 25 mM MES pH 7, 1 M NaCl, 5 mM BME). Ion-exchanged fractions containing only CBP TAZ2 were pooled, concentrated, flash frozen in liquid nitrogen and stored at -80°C until ready to use for GST-CTT-FLAG pull-downs; alternatively, pooled fractions were directly dialyzed into NMR Sample Buffer.

NMR spectroscopy

A 15N T2 relaxation experiment was acquired at 15°C on a 700 MHz spectrometer, equipped with a TXI probe. The experiment was recorded as a pseudo3D in an interleaved manned using the standard Bruker pulse sequence "hsqct2etf3gpsi3d". Relaxation delays of 16.3, 32.6, 65.2, 130.4, 163, 195.6, 228.2 and 260.8 ms were used; 32.6 ms was measured twice for error evaluation. An interscan delay of 5 sec was used and 20 scans were collected for each increment. 128 points were collected in the indirect ^{15}N dimension with a spectral width of 22 ppm, 1024 point were collected in the direct ^{1}H dimension with a spectral width of 16.2 ppm. The carrier was centered at 4.7 ppm in the ^{1}H dimension and at 117 ppm in the indirect ^{15}N dimension. Spectra were processed with NMRPipe[17] and the decay fitted CCPNmr[18]. Data analysis was performed excluding overlapping resonances.

Spectra were also recorded on an 800 MHz Bruker Advance spectrometer equipped with a TCI cryoprobe with z-shielded gradients and an Avance III console at 15°C. 3D-experiments were performed using NUS, collecting 10% of the Nyquist grid in the indirect dimension, using Poisson-Gap sampling. The resulting non-uniformly sampled spectra were reconstructed using the hmsIST algorithm[19]. Data were processed using NMRPipe[17] and analyzed with CCPNmr[18]. For residue assignment, a sample of 384 mM ^{15}N^{13}C-labeled His$_6$-mouse Aire CTT in NMR Sample Buffer supplemented with 5% D_2O was transferred into a 5 mm Shigemi tube. A 2D 15N-HSQC and a set of triple resonance assignment experiments (HNCA, HncoCA, HNCO, HncaCO, HNCACB, hCCCcoNH) were recorded. ^{15}N^{13}C-labeled His$_6$-mouse Aire CTT from the same preparation was mixed with unlabeled CBP TAZ2 and the complex was dialyzed in NMR Sample Buffer. A 2D 15N-HSQC and the same set of triple resonance assignment experiments mentioned above were run on this Aire:CBP sample (ratio 1:1.75). To assign the region showing exchange broadening, 50 mg urea (~1 M) was added in the ^{15}N^{13}C-labeled His$_6$-mouse Aire CTT. The experiments were repeated, leading to 98% assignment of non-proline (non-His$_6$-tag) residues. These assignments were then transferred to unbound His$_6$-mouse Aire CTT, resulting in assignment of 81% of non-proline residues.

References for Supplementary Note

1. Huoh, Y.S. *et al.* Dual functions of Aire CARD multimerization in the transcriptional regulation of T cell tolerance. *Nat Commun* **11**, 1625 (2020).

2. Lam, S.S. *et al.* Directed evolution of APEX2 for electron microscopy and proximity labeling. *Nat Methods* **12**, 51-54 (2015).

3. Maier, C.J., Maier, R.H., Hintner, H., Bauer, J.W. & Onder, K. Coupled yeast 2-hybrid-mammalian 2-hybrid reading-frame-independent and site-specific recombinational cloning vector system. *Assay Drug Dev Technol* **8**, 625-629 (2010).

4. Sanjana, N.E., Shalem, O. & Zhang, F. Improved vectors and genome-wide libraries for CRISPR screening. *Nat Methods* **11**, 783-784 (2014).

5. Nissim, L. *et al.* Synthetic RNA-Based Immunomodulatory Gene Circuits for Cancer Immunotherapy. *Cell* **171**, 1138-1150 e1115 (2017).

6. Askew, E.B., Bai, S., Blackwelder, A.J. & Wilson, E.M. Transcriptional synergy between melanoma antigen gene protein-A11 (MAGE-11) and p300 in androgen receptor signaling. *J Biol Chem* **285**, 21824-21836 (2010).

7. Wojciak, J.M., Martinez-Yamout, M.A., Dyson, H.J. & Wright, P.E. Structural basis for recruitment of CBP/p300 coactivators by STAT1 and STAT2 transactivation domains. *EMBO J* **28**, 948-958 (2009).

8. Wu, D., Wang, L. & Huang, H. Protocol to apply spike-in ChIP-seq to capture massive histone acetylation in human cells. *STAR Protoc* **2**, 100681 (2021).

9. Zhang, Y. *et al.* Model-based analysis of ChIP-Seq (MACS). *Genome biology* **9**, R137 (2008).

10. Ross-Innes, C.S. *et al.* Differential oestrogen receptor binding is associated with clinical outcome in breast cancer. *Nature* **481**, 389-393 (2012).

11. Robinson, M.D. & Oshlack, A. A scaling normalization method for differential expression analysis of RNA-seq data. *Genome biology* **11**, R25 (2010).

12. Whyte, W.A. *et al.* Master transcription factors and mediator establish super-enhancers at key cell identity genes. *Cell* **153**, 307-319 (2013).

13. Gilles, J.F., Dos Santos, M., Boudier, T., Bolte, S. & Heck, N. DiAna, an ImageJ tool for object-based 3D co-localization and distance analysis. *Methods* **115**, 55-64 (2017).

14. Cho, W.K. *et al.* Mediator and RNA polymerase II clusters associate in transcription-dependent condensates. *Science* **361**, 412-415 (2018).

15. Sabari, B.R. *et al.* Coactivator condensation at super-enhancers links phase separation and gene control. *Science* **361** (2018).

16. Yen, J.C., Chang, F.J. & Chang, S. A new criterion for automatic multilevel thresholding. *IEEE Trans Image Process* **4**, 370-378 (1995).

17. Delaglio, F. *et al.* NMRPipe: a multidimensional spectral processing system based on UNIX pipes. *J Biomol NMR* **6**, 277-293 (1995).

18. Vranken, W.F. *et al.* The CCPN data model for NMR spectroscopy: development of a software pipeline. *Proteins* **59**, 687-696 (2005).

19. Hyberts, S.G., Milbradt, A.G., Wagner, A.B., Arthanari, H. & Wagner, G. Application of iterative soft thresholding for fast reconstruction of NMR data non-uniformly sampled with multidimensional Poisson Gap scheduling. *J Biomol NMR* **52**, 315-327 (2012).
