# Supplementary material for: Mechanism for controlled assembly of transcriptional condensates by Aire
Source: Nat Immunol. 2024 Aug 21;25(9):1580–92. doi: 10.1038/s41590-024-01922-w (PMC11362013; doi:10.1038/s41590-024-01922-w)
Supplement: Supplementary file 2 — Reporting Summary [file 41590_2024_1922_MOESM2_ESM.pdf]

nature portfolio

Corresponding author(s): Sun Hur

Last updated by author(s): Jul 8, 2024

Reporting Summary

Nature Portfolio wishes to improve the reproducibility of the work that we publish. This form provides structure for consistency and transparency in reporting. For further information on Nature Portfolio policies, see our Editorial Policies and the Editorial Policy Checklist.

Statistics

For all statistical analyses, confirm that the following items are present in the figure legend, table legend, main text, or Methods section.

n/a	Confirmed	
☐	☒	The exact sample size (n) for each experimental group/condition, given as a discrete number and unit of measurement
☐	☒	A statement on whether measurements were taken from distinct samples or whether the same sample was measured repeatedly
☐	☒	The statistical test(s) used AND whether they are one- or two-sided<br>*Only common tests should be described solely by name; describe more complex techniques in the Methods section.*
☒	☐	A description of all covariates tested
☐	☒	A description of any assumptions or corrections, such as tests of normality and adjustment for multiple comparisons
☐	☒	A full description of the statistical parameters including central tendency (e.g. means) or other basic estimates (e.g. regression coefficient) AND variation (e.g. standard deviation) or associated estimates of uncertainty (e.g. confidence intervals)
☐	☒	For null hypothesis testing, the test statistic (e.g. F, t, r) with confidence intervals, effect sizes, degrees of freedom and P value noted<br>*Give P values as exact values whenever suitable.*
☒	☐	For Bayesian analysis, information on the choice of priors and Markov chain Monte Carlo settings
☒	☐	For hierarchical and complex designs, identification of the appropriate level for tests and full reporting of outcomes
☐	☒	Estimates of effect sizes (e.g. Cohen's d, Pearson's r), indicating how they were calculated

Our web collection on statistics for biologists contains articles on many of the points above.

Software and code

Policy information about availability of computer code

Data collection	No custom computer codes were used. Please see Methods section for details of all modes of data collection for experiments.
Data analysis	No custom computer codes were used. Please see Methods section for details of all data analyses performed for experiments.

For manuscripts utilizing custom algorithms or software that are central to the research but not yet described in published literature, software must be made available to editors and reviewers. We strongly encourage code deposition in a community repository (e.g. GitHub). See the Nature Portfolio guidelines for submitting code & software for further information.

Data

Policy information about availability of data

All manuscripts must include a data availability statement. This statement should provide the following information, where applicable:

- Accession codes, unique identifiers, or web links for publicly available datasets
- A description of any restrictions on data availability
- For clinical datasets or third party data, please ensure that the statement adheres to our policy

The accession numbers for the next generation sequencing data reported in this paper is Gene Expression Omnibus: GSE243825. Any additional information required to reanalyze the data reported in this paper is available from the lead contact corresponding author upon request.

Research involving human participants, their data, or biological material

Policy information about studies with human participants or human data. See also policy information about sex, gender (identity/presentation), and sexual orientation and race, ethnicity and racism.

Reporting on sex and gender	*Use the terms sex (biological attribute) and gender (shaped by social and cultural circumstances) carefully in order to avoid confusing both terms. Indicate if findings apply to only one sex or gender; describe whether sex and gender were considered in study design; whether sex and/or gender was determined based on self-reporting or assigned and methods used. Provide in the source data disaggregated sex and gender data, where this information has been collected, and if consent has been obtained for sharing of individual-level data; provide overall numbers in this Reporting Summary. Please state if this information has not been collected. Report sex- and gender-based analyses where performed, justify reasons for lack of sex- and gender-based analysis.*
Reporting on race, ethnicity, or other socially relevant groupings	*Please specify the socially constructed or socially relevant categorization variable(s) used in your manuscript and explain why they were used. Please note that such variables should not be used as proxies for other socially constructed/relevant variables (for example, race or ethnicity should not be used as a proxy for socioeconomic status). Provide clear definitions of the relevant terms used, how they were provided (by the participants/respondents, the researchers, or third parties), and the method(s) used to classify people into the different categories (e.g. self-report, census or administrative data, social media data, etc.) Please provide details about how you controlled for confounding variables in your analyses.*
Population characteristics	*Describe the covariate-relevant population characteristics of the human research participants (e.g. age, genotypic information, past and current diagnosis and treatment categories). If you filled out the behavioural & social sciences study design questions and have nothing to add here, write "See above."*
Recruitment	*Describe how participants were recruited. Outline any potential self-selection bias or other biases that may be present and how these are likely to impact results.*
Ethics oversight	*Identify the organization(s) that approved the study protocol.*

Note that full information on the approval of the study protocol must also be provided in the manuscript.

Field-specific reporting

Please select the one below that is the best fit for your research. If you are not sure, read the appropriate sections before making your selection.

☒ Life sciences ☐ Behavioural & social sciences ☐ Ecological, evolutionary & environmental sciences

For a reference copy of the document with all sections, see nature.com/documents/nr-reporting-summary-flat.pdf

Life sciences study design

All studies must disclose on these points even when the disclosure is negative.

Sample size	Sample sizes varied depending on experiments and are noted in the figure legends and Methods.
Data exclusions	For microscopy experiments examining Aire localization, we excluded cells that did not appear to express Aire (cells with fluorescence signals dimmer than background levels). For next-generation sequencing analyses, please see Methods for details of excluding background noises and blacklisted regions.
Replication	All experiments were performed at least 3 times. For qPCR data, in addition to 3 biological replicates, 2-3 technical replicates were performed. Only experimental data that were successfully replicated in all attempts are reported. See Figure legends for details.
Randomization	For microscopy, images were taken at random locations on the cover slips. For RNA-FISH coupled to IF experiments, random nuclear positions were generated to determine whether Aire foci was significantly associated with RNA FISH spots. Other experiments in this study were not subjected to randomization as the identity of the samples are predetermined during experiments; the experimental results would not be interpretable if these samples were randomized.
Blinding	For microscopy of cells treated with p300/CBP inhibitors or BRD4 inhibitors, samples were blinded while imaging. For all other experiments, there were no samples that could be blinded as the identity of the samples are predetermined during experiments.

Reporting for specific materials, systems and methods

We require information from authors about some types of materials, experimental systems and methods used in many studies. Here, indicate whether each material, system or method listed is relevant to your study. If you are not sure if a list item applies to your research, read the appropriate section before selecting a response.

Materials & experimental systems

n/a	Involved in the study	
☐	☒	Antibodies
☐	☒	Eukaryotic cell lines
☒	☐	Palaeontology and archaeology
☒	☐	Animals and other organisms
☒	☐	Clinical data
☒	☐	Dual use research of concern
☒	☐	Plants

Methods

n/a	Involved in the study	
☐	☒	ChIP-seq
☐	☒	Flow cytometry
☒	☐	MRI-based neuroimaging

Antibodies

Antibodies used

Antibodies used for immunofluorescence (IF) microscopy were mouse anti-FLAG (M2, Sigma-Aldrich, F1804), mouse anti-FLAG conjugated with FITC (M2, Sigma-Aldrich, F4049), rabbit anti-p300 (D8Z4E, Cell Signaling Technology, 86377S), rabbit anti-CBP (D6C5, Cell Signaling Technology, 7389S), rabbit anti-MED1 (Novus Biologicals, NB100-2574), Alexa Fluor® 488 AffiniPure donkey anti-mouse IgG (Jackson ImmunoResearch, 715-545-151), Alexa Fluor 647 AffiniPure donkey anti-rabbit IgG (Jackson ImmunoResearch, 711-605-152). Antibodies used for immunoblotting were rabbit anti-beta-actin (Cell Signaling Technology, 8457S), rabbit anti-HA (C29F4, Cell Signaling Technology, 3724S), mouse anti-FLAG-HRP (M2, Sigma-Aldrich, A8592), mouse anti-Lamin A (133A2, Cell Signaling Technology, 86846), mouse anti-Histone H3 (Cell Signaling Technology, 14269S), rabbit anti-Histone H3K27ac (D5E4, Cell Signaling Technology, 8173S), rabbit anti-Histone H3K18ac (D8Z5H, Cell Signaling Technology, 13998), rabbit anti-p300 (D8Z4E, Cell Signaling Technology, 86377S), rabbit anti-CBP (D6C5, Cell Signaling Technology, 7389S), rabbit anti-actyl-p300/CBP (Cell Signaling Technology, 4771S), anti-rabbit IgG-HRP (Cell Signaling Technology, 7074), anti-mouse IgG-HRP (Cell Signaling Technology, 7076). Antibodies used for chromatin immunoprecipitation mouse anti-FLAG (M2, Sigma-Aldrich, F1804), rabbit anti-Histone H3K27ac (D5E4, Cell Signaling Technology, 8173S), rabbit anti-Histone H3K4me1 (D1A9, Cell Signaling Technology, 5326S), rabbit anti-Histone H3K27me3 (C36B11, Cell Signaling Technology, 9733S), rabbit anti-Histone H3K4me0 (Active Motif, 91317), rabbit anti-p300 (D2X6N, Cell Signaling Technology, 54062), rabbit anti-BRD4 (E2A7X, Cell Signaling Technology, 13440), spike-in antibody (Active Motif, 61686).

Validation

All primary antibodies were validated previously by the manufacturer. Citations of studies using these antibodies and user ratings are provided on the manufacturer's websites

Eukaryotic cell lines

Policy information about cell lines and Sex and Gender in Research

Cell line source(s)

Human embryonic kidney cells 293T were a generous gift Dr. Dan Stetson, University of Washington; Seattle, WA. 4D6 cells were originally derived from human thymic epithelium from children undergoing cardiac surgery and provided to the laboratory of Diane Mathis.

Authentication

No form of authentication was used for these cell lines.

Mycoplasma contamination

These cells were verified to be mycoplasma free by using the MycoAlert Mycoplasma Detection Kit (Lonza, Cat. No. LT07-318).

Commonly misidentified lines
(See ICLAC register)

Unfortunately, we were not aware that 4D6 cell line was on the "Cross Contaminations Distribution List" as contaminated with human liver carcinoma cell line until we saw version 13 from April 2024 in the Author Guidance. We received these as a gift from Dr. Diane Mathis, who's lab has used this cell line routinely to study Aire. Many of our results have corroborated with the Mathis lab's previous findings and can be recapitulated in other cell lines including 293T.

Plants

Seed stocks

Report on the source of all seed stocks or other plant material used. If applicable, state the seed stock centre and catalogue number. If plant specimens were collected from the field, describe the collection location, date and sampling procedures.

Novel plant genotypes

Describe the methods by which all novel plant genotypes were produced. This includes those generated by transgenic approaches, gene editing, chemical/radiation-based mutagenesis and hybridization. For transgenic lines, describe the transformation method, the number of independent lines analyzed and the generation upon which experiments were performed. For gene-edited lines, describe the editor used, the endogenous sequence targeted for editing, the targeting guide RNA sequence (if applicable) and how the editor was applied.

Authentication

Describe any authentication procedures for each seed stock used or novel genotype generated. Describe any experiments used to assess the effect of a mutation and, where applicable, how potential secondary effects (e.g. second site T-DNA insertions, mosiacism, off-target gene editing) were examined.

ChIP-seq

Data deposition

☒ Confirm that both raw and final processed data have been deposited in a public database such as GEO.

☒ Confirm that you have deposited or provided access to graph files (e.g. BED files) for the called peaks.

Data access links<br>*May remain private before publication.*	The accession numbers for the ChIP-seq data reported in this paper is Gene Expression Omnibus: GSE243825
Files in database submission	Raw and processed data (bigwig) were uploaded.
Genome browser session<br>(e.g. UCSC)	No longer applicable.

Methodology

Replicates	All experiments were performed 2-3 times.
Sequencing depth	Pair-end 150bp reads, 30-40M reads per sample.
Antibodies	mouse anti-FLAG (M2, Sigma-Aldrich, F1804), rabbit anti-Histone H3K27ac (D5E4, Cell Signaling Technology, 8173S), rabbit anti-Histone H3K4me1 (D1A9, Cell Signaling Technology, 5326S), rabbit anti-Histone H3K27me3 (C36B11, Cell Signaling Technology, 9733S), rabbit anti-Histone H3K4me0 (Active Motif, 91317), rabbit anti-p300 (D2X6N, Cell Signaling Technology, 54062), spike-in antibody (Active Motif, 61686).
Peak calling parameters	Peak calling was performed using MACS2 (v2.2.7.1) with the following parameters: macs2 callpeak -f BAMPE -B -g 3.2e+9 --keep-dup 1 --SPMR --nomodel --extsize 250 -q 0.05 --cutoff-analysis. See Methods for more details.
Data quality	Alignments with MAPQ>1 were filtered. Replicates were examined to ensure reproducibility. Peaks were called using the filter FDR<0.05.
Software	FASTQC (v0.11.3), Trimmomatic (v0.36), deepTools (v3.5.1), WiggleTools (v1.2.2), bedGraphToBigWig (v366), Integrative Genomics Viewer (IGV, v2.15.1), bwa (v0.7.17), Samtools (v1.6), MACS2 (v2.2.7.1), DiffBind R package (v3.8.4)

Flow Cytometry

Plots

Confirm that:

☒ The axis labels state the marker and fluorochrome used (e.g. CD4-FITC).

☒ The axis scales are clearly visible. Include numbers along axes only for bottom left plot of group (a 'group' is an analysis of identical markers).

☒ All plots are contour plots with outliers or pseudocolor plots.

☒ A numerical value for number of cells or percentage (with statistics) is provided.

Methodology

Sample preparation	Cells were treated with 1 µg/ml Dox 24 hrs prior to sorting or acquisition to induce EGFP-P2A-Gal4DBD-AireCTT expression. In some experiments, cells were also treated with DMSO, A-485 (3 µM), or dCBP-1 (0.25 µM) for 24hrs prior to acquisition.
Instrument	Cells for CRISPR screening were sorted on SH800S Cell Sorter (Sony Biotechnology). Cells for flow cytometric analyses were acquired on FACSCanto II-AR (BD Biosciences).
Software	Flow cytometric data were analyzed using FlowJo (v10).
Cell population abundance	>98% cells were EGFP+ (Gal4DBD-AireCTT+) post 24 hrs of Dox treatment.
Gating strategy	Cells sorted for CRISPR screening were first gated on singlets based on FSC/SSC, and then gated on live (DAPI–) EGFP+ cells; top-5% and bottom-5% of the population were sorted based on mKate2 expression. Cells for flow cytometric analyses were first gated on singlets based on FSC/SSC.

☒ Tick this box to confirm that a figure exemplifying the gating strategy is provided in the Supplementary Information.
